# Husbandry Practices, Breeding Systems, and Reproductive Performance of Indigenous Sheep Under Smallholder Farming Systems in Loma District, Dawro Zone, Southwestern Ethiopia

**DOI:** 10.1155/tswj/3703823

**Published:** 2026-10-20

**Authors:** Abayneh Minota, Asrat Guja, Nebiyu Yemane, Kebede Gelgelo

**Affiliations:** ^1^ Department of Animal Sciences College of Agricultural Sciences Arba Minch University Arba Minch Ethiopia amu.edu.et

**Keywords:** breeding objectives, Dawro, husbandry practices, ram selection, reproductive performance

## Abstract

Comprehensive understanding of farmers′ breeding goals and reproductive performance of sheep is a foundational prerequisite in designing successful breeding strategies for smallholder systems. This study characterized sheep husbandry, breeding management, and reproductive performance among smallholder households in Loma District, Dawro Zone. A cross‐sectional survey of 138 sheep‐owning households was complemented by focus group discussions, key informant interviews, and field observations. Data were analyzed using descriptive statistics, ANOVA, chi‐square tests, ranking indices, and qualitative triangulation. Results showed that sheep production was multifunctional, with income generation and risk management receiving the highest importance among the identified purposes. Natural pasture was the dominant feed resource, whereas improved forage and nonconventional feeds were used less frequently. Natural mating was the principal breeding approach practiced by the farmers; none of them used artificial insemination. Selection of breeding rams was widely practiced by farmers irrespective of agroecological differences, where the ram was sourced from neighbors and from the replacement flock. Farmers focus on body conformation (34.1%) and age (26.8%), considering pedigree (10.9%) and coat color (9.4%) less frequently. Disease and parasites (index = 0.18), feed and water shortages (0.18), and weak extension (0.16) were the most severe bottlenecks of sheep herding; marketing problems, drought, and lack of credit services were the least limiting ones. The AFS (age at first service) of rams and ewes was noted to be 6.55 ± 0.91 and 7.28 ± 1.21 months, respectively, whereas AFL (age at first lambing), LI (lambing interval), and SAR (slaughter age of ram) were 12.30 ± 0.93, 7.67 ± 1.28, and 8.11 ± 0.84 months, respectively. The findings indicate that improvement efforts in Loma should build on existing farmer practices while strengthening feed availability, animal health services, farmer training, community‐based ram selection, and extension support.

## 1. Introduction

According to the recent CSA report, Ethiopia had one of the largest livestock populations in Africa and globally, comprising about 61.5 million cattle, 33 million sheep, 38.9 million goats, 1.76 million camels, and more than 59.4 million poultry [1]. Among the livestock sectors, the sheep production industry was renowned to be economically important [2–4]. The subsector is a multifaceted and indispensable component of the agricultural sector, deeply embedded in the socioeconomic fabric of smallholder pastoral, agropastoral, and mixed crop‐livestock rearing families by providing a critical source of income, meat, manure, and sociocultural value [5].

Besides their indispensable role, their higher adaptability to different climatic conditions, faster growth and reproductive rates, lower capital and input costs, ease of care, and feeding relative to large ruminants make them more advantageous and preferable for the low‐income smallholder farming systems [6, 7]. In Ethiopia, indigenous sheep breeds dominate the national sheep population, accounting for approximately 99.5%–99.9% of the total sheep population, whereas exotic breeds constitute only a negligible proportion. This highlights the indispensable role of indigenous sheep breeds in the national economy and in sustaining the livelihoods of smallholder sheep‐producing communities [1].

Despite its importance, the sheep production industry faces significant constraints, including chronic feed shortages, high disease incidence, poor extension services, lack of credit services, limited genetic improvement, and inefficient marketing channels [5]. In order to improve the overall productivity and contribution of sheep to livelihoods and poverty reduction in smallholder operations, identification of the production constraints and searching for possible interventions to address the identified constraints are highly recommended [2, 4]. In this regard, few efforts have been made with different breeds over the years in different parts of Ethiopia [8–10]. Nevertheless, the study of different breeds will help unlock more information on the productive and reproductive potential of indigenous sheep, which are widely distributed in the different agroecological areas throughout the country [11]. Moreover, a comprehensive understanding of the existing sheep‐rearing operations and farmers′ breeding practices is a foundational prerequisite for successful implementation of breeding objectives designed for any kind of sheep‐herding community [10]. Limited empirical information exists regarding sheep husbandry practices, breeding systems, and reproductive performance in Loma District, constraining evidence‐based genetic improvement and extension planning. Therefore, this study was conducted to explore the husbandry practices, constraints, breeding systems, and reproductive performance of local sheep in Loma District of Dawro Zone, Southwestern Ethiopia.

## 2. Materials and Methods

### 2.1. Description of the Study Area

The study was conducted in Loma District of Dawro Zone, Southwestern Regional State, Ethiopia. Gessa is the capital town of the district, located about 17 km from Tarcha, the zonal town; 249 km from Bonga, the regional town; and 457 km from Addis Ababa, the capital city of Ethiopia. Loma is located at 6°50 ^′^0 ^″^–7°6 ^′^0 ^″^ N latitude and 37°10 ^′^0 ^″^–37°26 ^′^0 ^″^ E longitude, approximately 501–2800 m above sea level [12]. The district is divided into 25 rural kebeles and 2 municipalities. The total land area is 56,340 ha, out of which 21,424.5 ha is cultivated cropland, 16,529.5 ha is covered with perennial crops, 1797.125 ha is grazing land, 4809 ha is forest land, 10,881 ha is covered with bushes and undulating land, and 898.875 ha is for urban construction purposes. The average temperature is 22°C, with minimum and maximum temperatures of 14°C and 30°C, respectively. Rainfall in the district occurs across two main periods: the long rainy season (June–September), the short rainy season (March–April) while dry season occures between October to May. The mean annual rainfall is 1046 mm and ranges from 780 to 1312 mm. *Belg* and *Meher* are the two cropping seasons, with the short and long rainy periods, respectively. The economy of the district is mainly based on mixed agriculture, including both crop and livestock production [13]. A map of the study area is presented in Figure 1.

**Figure 1:**
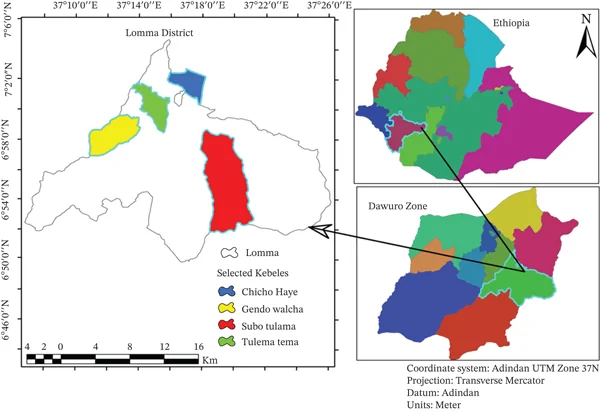
Map of the study area.

### 2.2. Sample Size Determination and Sampling Techniques

The sample size was determined using the formula recommended by Cochran [14]:

n=Z2p qd21.962 0.10.90.052=138,

where *n* = sample size, *Z* ^2^ = value of selected alpha level (1.96 corresponding to 95% confidence interval), *p* = estimated proportion of an attribute that is present in the population (10%), *q* = estimate of variance (1 − *p* = 0.9), and *d* ^2^ = acceptable margin of error for proportion being estimated (5%).

Therefore, 138 respondents were selected randomly.

For sampling purposes, Loma District was classified into three agroecological zones according to altitude: highland, midland, and lowland. Four kebeles (the lowest administrative unit in Ethiopia) were subsequently included in the survey, comprising two highland kebeles, one midland kebele, and one lowland kebele. The kebeles were selected purposively based on their sheep production potential. Household lists obtained from the respective agricultural offices served as the sampling frames, from which sheep‐owning households were selected randomly. The final sample comprised 72 households from the highland, 34 from the midland, and 32 from the lowland agroecological zones.

### 2.3. Methods of Data Collection

#### 2.3.1. Field Survey

A structured questionnaire was designed and translated into the local language (*Dawurtho*) of the study community. Development agents (DAs) who were working in the district and who spoke the local language were trained and assigned to collect the data under close supervision of the researchers. Data on variables like sociodemographic characteristics of households, purposes of keeping sheep, husbandry practices, feeds, water sources and watering frequencies, breeding system and ram selection, reproductive performance, and constraints of sheep herding were collected.

#### 2.3.2. Focus Group Discussion (FGD)

FGD was arranged with 10–12 members, including elders, village leaders, agricultural extension workers, and model farmers who have good experience of sheep production. A checklist generated from the questionnaire was used to guide the FGD. The information obtained from the FGD was used to complement the survey work.

#### 2.3.3. Key Informant Interview

Primary data were also generated by formal interviews with key informants like agricultural officers of the study kebeles and district. The key informant interviews were conducted using interview guiding questions derived from the structured questionnaire, intending to complement and validate the questionnaire and FGD data.

#### 2.3.4. Field Observation

During questionnaire administration, production practices, available feed resources, and other relevant information were personally observed during field visits.

### 2.4. Data Management and Statistical Analysis

Data were analyzed using one‐way ANOVA procedures in SPSS software (Version 26). Means were separated using Duncan multiple range test if the differences between means were declared significant at *p* < 0.05. Chi‐square tests of independence were performed for categorical data satisfying chi‐square assumptions. For those categorical data violating chi‐square assumptions, Fisher′s exact test was performed. The statistical model used for quantitative data analysis was as follows:

Yij=μ+αi+Ɛij,

where *Y*_*i* *j*_ = response variable, *μ* = overall mean, *α*_*i*_ = fixed effect of agroecology (*i* = 3; highland, midland, and lowland), and *Ɛ*_*i* *j*_ = random error.

Normality was tested via the Shapiro–Wilk test, whereas homogeneity of variance was tested using Levene′s test. Ranking data were analyzed by indices computed with the principle of weighted average, and indices were ranked against each other using autoranking. The following formula was used to compute the ranking index according to Musa et al. [15]:

Index=Rn∗C1+Rn−1∗C2⋯+R1∗Cn∑Rn∗C1+Rn−1∗C2⋯+R1∗Cn,

where *R*_*n*_ = value given for the least ranked level (e.g., if the least rank is fifth, then *R*_*n*_ = 5, *R*_*n*−1_ = 4, and *R*_1_ = 1) and *C*_*n*_ = count of the least ranked level (in the above example, the count of the fifth rank is *C*_*n*_, and the count of the first rank is *C*_1_).

## 3. Results and Discussion

### 3.1. Sociodemographic Characteristics

In the study area, 71.0% of the interviewed sheep producers were males, and the remaining 29% were females, with no association between sex of the household and agroecology (*p* > 0.05) (Table 1). This result revealed that the majority of sheep producers in the area were male‐headed. This finding was consistent with that of Afras et al. [16], who reported 76.8% male‐ and 23.2% female‐headed households in Esera District, Dawro Zone, Southern Ethiopia, and Debir and Amelmal [9], who reported 82% male‐ and 18% female‐headed small ruminant keeping households in the Gedeb District of the Gedeo Zone, Southern Ethiopia. Disparity in gender proportion may imply limited power of women in decision‐making regarding sheep production, warranting gender‐inclusive livestock development programs in the area. Respondents aged between 30 and 39 years dominated across all agroecologies, with an overall share of 47.1%, followed by those aged between 40 and 50 years, with respondents above 50 years representing the smallest proportion. The dominance of the young and productive age group in sheep husbandry was a very common observation across different regions of Ethiopia [2, 8, 17]. The dominance of younger and active age groups suggests strong potential for adoption of improved sheep production technologies and breeding interventions.

**Table 1:** Sociodemographic profile of households among the respondents.

Variables	Description	Agroecology			Total *N* (%)	Chi‐square	*p* value
		Highland *N* (%)	Midland *N* (%)	Lowland *N* (%)			
Sex	Male	53 (73.61)	21 (61.76)	24 (75.00)	98 (71.01)	1.896	0.388
	Female	19 (26.39)	13 (38.24)	8 (25.00)	40 (28.99)		
	Total	72 (100)	34 (100)	32 (100)	138 (100)		

Age	18–29	16 (22.22)	8 (23.53)	5 (15.63)	29 (21.01)	5.826	0.443
	30–39	37 (51.39)	15 (44.12)	13 (40.62)	65 (47.10)		
	40–50	15 (20.83)	11 (32.35)	12 (37.50)	38 (27.54)		
	> 50	4 (5.56)	0 (0.00)	2 (6.25)	6 (4.35)		
	Total	72 (100)	34 (100)	32 (100)	138 (100)		

Marital status	Married	60 (83.33)	26 (76.47)	31 (96.88)	117 (84.78)	12.628	0.049
	Single	10 (13.89)	3 (8.82)	0 (0.00)	13 (9.42)		
	Divorced	1 (1.39)	3 (8.82)	0 (0.00)	4 (2.90)		
	Widowed	1 (1.39)	2 (5.88)	1 (3.12)	4 (2.90)		
	Total	72 (100)	34 (100)	32 (100)	138 (100)		

Family size	1–3	24 (33.33)	9 (26.47)	10 (31.25)	43 (31.16)	14.194	0.007
	4–6	39 (54.17)	17 (50.00)	8 (25.00)	64 (46.38)		
	7–9	9 (12.50)	8 (23.53)	14 (43.75)	31 (22.46)		
	Total	72 (100)	34 (100)	32 (100)	138 (100)		

Educational level	Illiterate	20 (27.78)	12 (35.29)	15 (46.88)	47 (34.05)	16.304	0.091
	Read and write	5 (6.94)	9 (26.47)	3 (9.37)	17 (12.32)		
	Primary school	16 (22.22)	4 (11.76)	5 (15.62)	25 (18.12)		
	Junior primary school	14 (19.44)	5 (14.71)	6 (18.75)	25 (18.12)		
	Secondary school	12 (16.67)	2 (5.88)	3 (9.38)	17 (12.32)		
	College and above	5 (6.94)	2 (5.88)	0 (0.00)	7 (5.07)		
	Total	72 (100)	34 (100)	32 (100)	138 (100)		

According to the findings of this study, 84.8% of the respondents were married, 9.4% were single, 2.9% were widowed, and 2.9% were divorced, with marital status having a significant association (*p* < 0.05) with agroecology. This was consistent with the observations of Dekamo et al. [2], who reported higher proportions of married respondents (98.3%) among sheep producers in Sidama Region, Southern Ethiopia. In the present study, respondents with a family size of four to six persons per household dominated in highland and midland areas, whereas farmers with seven to nine persons per household dominated in the lowlands. In accordance with this, higher family sizes, usually five to nine persons per household, were most frequently noted among small ruminant keepers across various corners of Ethiopia [8, 18].

Illiterate households were the dominant (34.1%) category of the sheep‐producing community, whereas those who attended tertiary education (college and above) were the least (5.1%). Varying illiteracy rates were reported by different authors for the sheep‐rearing community in different regions of Ethiopia. Dekamo et al. [2] reported 25.8% illiteracy in different districts of Sidama Region, Southern Ethiopia, lower than the illiteracy of the present study. On the contrary, Debir and Amelmal [9] noted 54% illiteracy for a sheep‐rearing community in the Gedeb District of the Gedeo Zone of Southern Ethiopia which is by far higher than the illiteracy of the present study. The inconsistency in the illiteracy rate across different localities of sheep‐producing communities could imply a discrepancy in accessibility to education among the sheep‐rearing communities which could meaningfully influence technology transfer and development levels among the farmers. In this regard, Moreno‐Monroy et al. [19] argued that accessibility to schools and educational services was positively correlated with the literacy level and development of a community. Besides, education is widely recognized as a foundational tool for community empowerment and long‐term development, serving as a critical entry point for improving both individual livelihoods and collective community resilience. By equipping individuals with knowledge, critical thinking, and new skills, education acts as a catalyst for breaking cycles of poverty and enhancing the socioeconomic status of families [20, 21]. According to Gebre [22], higher educational levels of a household lead to decreased family sizes. This could explain the higher illiteracy rate and higher family size noted in the lowlands of the study area in the present study. In the present study, the higher proportion of married households in the lowlands with relatively higher illiteracy is consistent with studies indicating that individuals with limited educational opportunities often enter marriage at younger ages than their more educated counterparts [23].

### 3.2. Landholding Patterns

Table 2 summarizes the landholding patterns among the sheep herders in the study area. Significant variability was observed among the households in holding patterns of all land types (*p* < 0.05) across agroecology except grazing land (*p* > 0.05). Cropland as well as total landholding size showed a consistent pattern of variation across agroecologies, with land holdings following the order: highlands < midlands < lowlands (*p* < 0.001). On the other hand, farmers in lowland areas had improved forage land comparable to those of highlands but higher than midlands (*p* < 0.05). In Bensa District of Sidama Region, Southern Ethiopia, Kenfo et al. [24] reported grazing land (0.3 ha) nearly comparable to the present study (0.28 ha), cropland (1.1 ha) lower than the present study (1.21 ha), but total land (2.13 ha) higher than the present study (1.62 ha). The total landholding per household observed in the study area was also slightly higher than the national average household land size of rural Ethiopia (1.1 ha) reported by FAO [25] but considerably lower than 3.12 ha reported by Afras et al. [16] in Esera District, Dawro Zone. Access to and control of land is the absolute foundation of any successful livestock improvement package. Without a dedicated, secure land base, interventions like breed upgrades, intensive feeding, and veterinary services will struggle to succeed [26]. So inconsistency in landholding across agroecology could suggest that livestock‐related packages designed in the area should not be generic and should be tailored to agroecological settings. The observed variation in crop and total landholding suggests that the feasibility of forage establishment and other livestock interventions may differ among agroecological zones. Consequently, interventions requiring dedicated land resources should be evaluated against the land availability of individual production environments.

**Table 2:** Landholding per household in hectares (mean ± SD [standard deviation]).

Land type (hectare)	Agroecology			Overall	SEM	*p* value
	Highland	Midland	Lowland			
Grazing land	0.21 ± 0.41^a^	0.32 ± 0.48^a^	0.41 ± 0.49^a^	0.28 ± 0.45	0.038	0.099
Cropland	0.81 ± 0.39^c^	1.32 ± 0.48^b^	2.00 ± 0.51^a^	1.21 ± 0.66	0.056	< 0.001
Improved forage land	0.11 ± 0.32^ab^	0.06 ± 0.24^b^	0.22 ± 0.42^a^	0.12 ± 0.33	0.028	0.048
Total landholding	1.13 ± 0.33^c^	1.71 ± 0.63^b^	2.63 ± 0.71^a^	1.62 ± 0.79	0.068	< 0.001

*Note:* Means with different superscript letters (a, b, and c) across rows varied significantly.

Abbreviation: SEM, standard error of the mean.

### 3.3. Flock Structure and Composition

The sheep flock structure and composition in the study area are presented in Table 3. The average number of sheep types varied significantly among the households with varying agroecologies (*p* < 0.05). Lowland farmers had fewer lambs compared with highland and midland ones (*p* < 0.001). The average number of ram lambs, ewe lambs, and breeding ewes varied consistently, following the increasing order: highlands < midlands < lowlands (*p* < 0.001). Castrated rams of midland farmers were relatively higher than those of lowlands but comparable to those of highlander families (*p* > 0.05). Generally, the highest and lowest observations of total flock were noted in highlands and lowlands, respectively, whereas intermediate values of total flock size were recorded for midland families. Location‐based variability in sheep flock was frequently noted by earlier reports, but the average flock holding was not consistent among these and other studies. In Northern Ethiopia, Tsigab et al. [27] reported average numbers of lambs (10.98), ram lambs (2.35), ewe lambs (11.67), breeding ewes (29.26), and breeding rams (1.63) higher than those in the present study, but castrates (0.02) lower than in the present study (0.16). On the other hand, Debir and Amelmal [9] reported higher number of lambs (2.21), breeding ewes (3.8), ewe lambs (2.1) and total flock (8.51) than the records of the present study, but lower number of breeding rams (0.2) and ram lambs (0.3) than the breeding rams (0.42) and ram lambs (1.20) noted in the present study for sheep herders in Gedeb district of Gedeo zone in southern Ethiopia. The observed inconsistency in flock structure and composition among this study and literature reports could be ascribed to environmental factors, landholdings, and feed availability dynamics as well as the variability in economic and management considerations across different study localities [10, 28].

**Table 3:** Flock structure and composition in head (mean ± SD [standard deviation]).

Sheep type	Agroecology			Overall	Proportion (%)	SEM	*p* value
	Highland	Midland	Lowland				
Lambs (< 6 months)	0.58 ± 0.55^a^	0.38 ± 0.49^a^	0.13 ± 0.36^b^	0.43 ± 0.53	7.40	0.045	< 0.001
Ram lambs (6–12 months)	1.47 ± 0.60^a^	1.18 ± 0.46^b^	0.63 ± 0.49^c^	1.20 ± 0.64	20.65	0.055	< 0.001
Ewe lambs (6–12 months)	1.83 ± 0.61^a^	1.18 ± 0.46^b^	0.78 ± 0.42^c^	1.43 ± 0.69	24.61	0.059	< 0.001
Breeding ewes (> 12 months)	2.64 ± 0.61^a^	2.29 ± 0.63^b^	1.00 ± 0.00^c^	2.17 ± 0.85	37.35	0.073	< 0.001
Breeding rams (> 12 months)	0.56 ± 0.53^a^	0.32 ± 0.48^b^	0.22 ± 0.42^b^	0.42 ± 0.51	7.23	0.043	0.003
Castrated ram	0.18 ± 0.39^ab^	0.24 ± 0.43^a^	0.03 ± 0.18^b^	0.16 ± 0.37	2.75	0.031	0.048
Total flock	7.26 ± 1.52^a^	5.59 ± 1.58^b^	2.78 ± 0.75^c^	5.81 ± 2.78	100	0.194	< 0.001

*Note:* Means with different superscript letters (a, b, and c) across rows varied significantly.

Abbreviation: SEM, standard error of the mean.

Breeding ewes share the highest proportion of the total flock (37.35%), followed by ewe lambs (24.61%), whereas castrated rams took the least share in the flock (2.75%), followed by breeding rams (7.23%). In the Gedeb District of the Gedeo Zone, Southern Ethiopia, Debir and Amelmal [9] reported a significantly higher proportion (44.4%) of breeding ewes in the flock, followed by lambs less than 6 months and ewe lambs. However, breeding rams were significantly lower among the sheep flocks. This was in line with observations of the present study. According to the authors, the larger proportion of breeding ewes implies the production of more lambs, which in turn might increase the intensity of selection and marketable lambs. Likewise, other authors [2, 10] also disclosed similar observations in different regions of Ethiopia. The argument of Debir and Amelmal [9] which revealed that sheep herders mostly remove rams from the flock at early stages but are accustomed to retain female sheep in the flock as replacement stock, could explain the observed flock structure in the present and other similar studies. Whereas maintaining a larger female breeding population provides a strong footing for community‐based genetic improvement programs, the low number of breeding rams may increase the risk of mating among related animals if replacement and mating are not adequately managed.

### 3.4. Purpose of Keeping Sheep

The purpose of keeping/rearing sheep in the study area is described in Table 4. Sheep ownership served several livelihood functions in Loma District. Farmers primarily viewed sheep as a means of generating cash and buffering households against economic and production‐related shocks, whereas meat and manure represented additional benefits. The relative importance of these functions varied among agroecological zones, indicating that farmers′ production objectives were shaped by local livelihood conditions. Generally, income generation was the primary objective, followed by insurance, meat source, and manure production, whereas serving as a means of saving was identified as the least important production objective for sheep herders in the study area. In line with the observations of the present study, earlier reports disclosed that indigenous sheep in Ethiopia have a multipurpose role for smallholder farmers as a vital source of immediate cash income, serving as a “living bank” for smallholders to cover expenses like school fees and agricultural inputs. They are essential for household nutrition (meat), providing manure for soil fertility [2–4]. Apart from the production objectives targeted by sheep herders in the present study, sheep were reported to have sociocultural significance, being integral to religious festivals and social ceremonies, including weddings and births, and serving as gifts to strengthen social bonds or as a form of wealth and prestige [5, 27], underlining the versatile role played by sheep that could vary across different corners of Ethiopia.

**Table 4:** Purposes of keeping sheep, ranked by the respondents in the study area.

Agroecology	Purpose	Ranked (*N*)					Total	Weight	Index	Rank
		1st	2nd	3rd	4th	5th				
Highland	Sale/cash income	35	20	17	0	0	72	306	0.2833	1st
	Insurance	20	34	18	0	0	72	290	0.2685	2nd
	Meat	17	18	37	0	0	72	268	0.2481	3rd
	Saving	0	0	0	37	35	72	109	0.1009	4th
	Manure	0	0	0	35	37	72	107	0.0991	5th
	Total	72	72	72	72	72		1080	1.0000	

Midland	Sale/cash income	16	7	11	0	0	34	141	0.2686	1st
	Insurance	8	17	9	0	0	34	135	0.2571	2nd
	Manure	10	10	14	0	0	34	132	0.2514	3rd
	Meat	0	0	0	19	15	34	68	0.1295	4th
	Saving	0	0	0	15	19	34	49	0.0933	5th
	Total	34	34	34	34	34		525	1.0000	

Lowland	Insurance	17	9	6	0	0	32	139	0.2896	1st
	Sale/cash income	9	15	8	0	0	32	129	0.2688	2nd
	Saving	6	8	18	0	0	32	116	0.2417	3rd
	Meat	0	0	0	17	15	32	49	0.1021	4th
	Manure	0	0	0	15	17	32	47	0.0979	5th
	Total	32	32	32	32	32		480	1.0000	

Overall	Sale/cash income	60	42	36	0	0	160	576	0.2783	1st
	Insurance	45	60	33	0	0	138	564	0.2725	2nd
	Meat	17	18	37	36	30	138	370	0.1787	3rd
	Manure	10	10	14	50	54	138	286	0.1382	4th
	Saving	6	8	18	52	54	138	274	0.1324	5th
	Total	138	138	138	138	138	712	2070	1.0000	

### 3.5. Sheep Husbandry Practice

#### 3.5.1. Feed Resources and Feeding Practice

The major feed resources utilized by the sheep herders in the study area are summarized in Table 5.

**Table 5:** Source of sheep feeds ranked by the respondents.

Agroecology	Feed type	Ranked (*N*)							Total	Weight	Index	Rank
		1st	2nd	3rd	4th	5th	6th	7th				
Highland	Natural pasture	35	19	18	0	0	0	0	72	449	0.223	1st
	River and roadside grazing	20	34	18	0	0	0	0	72	434	0.215	2nd
	Crop aftermath	17	19	36	0	0	0	0	72	413	0.205	3rd
	Crop residues	0	0	0	33	15	13	11	72	214	0.106	4th
	Hay	0	0	0	16	37	10	9	72	204	0.101	5th
	NCF (*Atela*)	0	0	0	8	11	36	17	72	154	0.076	6th
	Improved forage	0	0	0	15	9	13	35	72	148	0.073	7th
	Total	72	72	72	72	72	72	72	504	2016	1.000	

Midland	Natural pasture	18	9	7	0	0	0	0	34	215	0.226	1st
	Crop aftermath	5	19	10	0	0	0	0	34	199	0.209	2nd
	River and roadside grazing	11	6	17	0	0	0	0	34	198	0.208	3rd
	NCF (*Atela*)	0	0	0	16	8	6	4	34	104	0.109	4th
	Improved forage	0	0	0	6	18	7	3	34	95	0.100	5th
	Hay	0	0	0	9	3	17	5	34	84	0.088	6th
	Crop residues	0	0	0	3	5	4	22	34	57	0.060	7th
	Total	34	34	34	34	34	34	34	238	952	1.000	

Lowland	Natural pasture	17	8	7	0	0	0	0	32	202	0.225	1st
	River and roadside grazing	7	15	10	0	0	0	0	32	189	0.211	2nd
	Crop aftermath	8	9	15	0	0	0	0	32	185	0.206	3rd
	NCF (*Atela*)	0	0	0	14	8	7	3	32	97	0.108	4th
	Hay	0	0	0	9	13	6	4	32	91	0.102	5th
	Crop residues	0	0	0	6	7	12	7	32	76	0.085	6th
	Improved forage	0	0	0	3	4	7	18	32	56	0.063	7th
	Total	32	32	32	32	32	32	32	224	896	1.000	0

Overall	Natural pasture	70	36	32	0	0	0	0	138	866	0.232	1st
	River and roadside grazing	38	55	45	0	0	0	0	138	821	0.220	2nd
	Crop aftermath	30	47	61	0	0	0	0	138	797	0.214	3rd
	Hay	0	0	0	34	53	33	18	138	379	0.102	4th
	NCF (*Atela*)	0	0	0	38	27	49	24	138	355	0.095	5th
	Crop residues	0	0	0	42	27	29	40	138	347	0.093	6th
	Improved forage	0	0	0	24	31	27	56	138	299	0.080	7th
	Total	138	138	138	138	138	138		828	3726	1.000	

Feed availability in Loma was dominated by locally accessible resources. Natural pasture received the highest overall ranking, followed by roadside and riverine grazing and crop aftermath. In contrast, improved forages and conserved feeds contributed relatively little to the feeding system. This pattern suggests that sheep production remains strongly dependent on freely available vegetation and crop‐derived resources, with limited adoption of deliberately cultivated or conserved feed resources. The low ranking of improved forage suggests that forage cultivation has not yet become an established component of sheep feeding in the sampled households. Consistent with this, similar observations were noted by earlier reports in different regions of Ethiopia [4, 16, 27]. Besides, the low utilization of hay and improved forages was supported by the observations of Begna et al. [29], who reported the contribution of the stated feed resources to share 5%–25% of the annual feed base, with significant variations in feeding practices across various corners of Ethiopia. On the other hand, the limited contribution of the improved forages could be attributed to minimal adoption of the forage species as a result of land scarcity, lack of improved forage seed/planting material, lack of awareness, and poor extension services across different localities of Ethiopia [30]. The low adoption of improved forages indicates substantial extension work to be done through forage promotion programs.

#### 3.5.2. Water Source and Watering Frequency

The main source of water and watering frequency of the sheep in the study area is denoted in Table 6. Rivers, springs, tap water, community ponds, and wells were the most common water sources identified in the study areas. Water access varied markedly with agroecological position (*p* < 0.001). Springs were the predominant source in the highland (37.5%) and midland (38.2%) sites, whereas river water dominated in the lowland sample (65.6%). This spatial pattern was statistically significant, indicating that sheep water management in Loma is closely associated with local availability of surface and groundwater resources. This finding was in line with findings of Afras et al. [16] and Assefa et al. [4], who reported river water, ponds, tap water, and wells as the main sources of water for sheep in Esera District, Dawro Zone, Southern Ethiopia, and in the selected districts of Majang Zone, Southwest Ethiopia, respectively.

**Table 6:** Water sources and watering frequency of sheep in the Loma District in Dawro Zone.

Water sources	Agroecology			Total *N* (%)	Chi‐square	*p* value
	Highland *N* (%)	Midland *N* (%)	Lowland *N* (%)			
River	21 (29.17)	11 (32.35)	21 (65.63)	53 (38.41)	56.566	*p* < 0.001
Tap water	19 (26.39)	8 (23.53)	2 (6.25)	29 (21.01)		
Spring	27 (37.50)	13 (38.24)	0 (0.00)	40 (28.99)		
Ponds	0 (0.00)	0 (0.00)	9 (28.13)	9 (6.52)		
Wells	5 (6.94)	2 (5.88)	0 (0.00)	7 (5.07))		
Total	72 (100)	34 (100)	32 (100)	138 (100)		
Watering frequency per day						
Once	38 (52.78)	22 (64.71)	28 (87.50)	88 (63.77)	11.577	0.003
Twice	34 (47.22)	12 (35.29)	4 (12.50)	50 (36.23)		
Total	72 (100)	34 (100)	32 (100)	138 (100)		

Across all agroecologies, the majority of the farmers were watering their flock once per day (63.8%), and the least proportion of the respondents (36.2%) were watering their sheep twice a day, with watering frequency showing significant association with agroecology (**p** < 0.001). Corresponding to this, Kenfo et al. [24] and Assefa et al. [4] outlined that the majority of sheep herders provided water for their flock once a day, followed by those watering twice a day in Bensa District of Sidama Zone, Southern Ethiopia, and in the Gambella Region, Southwestern Ethiopia, respectively. The authors also reported that a small but nonnegligible proportion of the respondents (6.25%–10.3%) were watering their flock once every 2 days, a watering scenario not observed in the present study. The differences among agroecological zones indicate that sheep‐watering practices are closely associated with the availability and accessibility of local water resources.

#### 3.5.3. Housing Systems and Cleaning Frequency

Table 7 summarizes the sheep housing systems and house cleaning frequency in the study area. The housing system is not consistent across agroecology. The majority of farmers in highlands (54.2%) and midlands (61.8%) keep their sheep in a family house with a separate barn with other animals, followed by those keeping sheep in a separate house from the family (23.3%–26.4%) and attaching the house to the main family house (14.7%–19.4%). Farmers building sheep in a separate house from the family took the least proportion (6.3%) in lowlands as opposed to other agroecologies. Sheep sharing similar houses with the family may improve security and management on one side, but could increase disease transmission risks if housing design is inadequate on the other side. No significant association was detected between housing system and agroecological zone. These observations are in accordance with the report of Kenfo et al. [24], who stated that approximately 75% of sheep herders in Bensa District of Sidama Region, Southern Ethiopia, keep their flock in the main house together with the family. In Gondar Town of Amhara Regional State, Northwest Ethiopia [17], and in the Majang Zone of Gambella Region, Southwest Ethiopia [4], the majority (64%–70%) of the farmers keep their sheep in houses separated from the main family houses, which contradicts the observation of the present study. The variation among Ethiopian studies may reflect differences in land availability, household resources, perceived security needs, climatic conditions, and local housing traditions [31]. Across agroecological zones, farmers cleaning the sheep houses once a day (80.4%) dominated over those cleaning once every 2 days (19.6%), with house cleaning frequency and agroecology revealing significant associations (*p* < 0.05). In Ararso District of Jarar Zone, Eastern Ethiopia, Ismail et al. [32] noted a situation contradicting those of the present study, as sheep herders who clean sheep houses once every 2 days (76.7%) dominate over those cleaning daily (11.1%) and once every 3 days (12.2%).

**Table 7:** Housing system and cleaning frequency of sheep in the Loma District in Dawro Zone.

Housing system	Agroecology			Overall *N* (%)	Chi‐square	*p* value
	Highland *N* (%)	Midland *N* (%)	Lowland *N* (%)			
In a family house with a separate barn with other animals	39 (54.16)	21 (61.76)	18 (56.25)	78 (56.52)	9.072	0.059
Separate house from family	19 (26.39)	8 (23.53)	2 (6.25)	29 (21.01)		
Attached to the main family house	14 (19.44)	5 (14.7)1	12 (37.50)	31 (22.47)		
Total	72 (100)	34 (100)	32 (100)	138 (100)		
Frequency of cleaning						
Once a day	52 (72.22)	30 (88.24)	29 (90.63)	111 (80.43)	6.522	0.039
Once every 2 days	20 (27.78)	4 (11.76)	3 (9.37)	27 (19.57)		
Total	72 (100)	34 (100)	32 (100)	138 (100)		

### 3.6. Breeding Systems, Ram Source, and Selection Criteria

Table 8 summarizes the breeding systems, ram source, and selection criteria set by the farmers in the study area. No significant association was detected between agroecology and the breeding system, ram sources, and selection criteria (*p* > 0.05). Use of artificial insemination (AI) for sheep breeding is totally lacking in the area, and all of the sheep herder respondents were using natural mating. Selection of the best ram for breeding is a very common practice in the area. All of the farmers in the highlands and midlands and the majority of the respondents in lowlands (84.4%) practice ram selection, with the practice lacking among a very small proportion of the farmers (15.6%) in the lowlands. A significant association was confirmed between ram selection and agroecology (*p* < 0.001). This remark was consistent with the observations of Abdilahi et al. [33], who disclosed that 93.7% of the smallholder farmers in the Awbarre District of the Fafen Zone, Somali Region, Ethiopia, were practicing ram selection for mating. The majority of the farmers obtain rams from both neighbors and their own flock (49.3%). Farmers using rams born from their own flock only (19.6%) and neighbors′ rams (18.1%) had comparable proportions in the study community, whereas those who purchase rams from the market were in the lowest proportion (13%). The prevailing farmer‐led ram selection practices provide a strong groundwork for community‐based breeding programs.

**Table 8:** Breeding systems, ram source, and selection criteria.

Variables	Agroecology			Overall *N* (%)	Fisher′s exact	Exact *p* value
	Highland *N* (%)	Midland *N* (%)	Lowland *N* (%)			
Breed system						
Using AI	0 (0.00)	0 (0.00)	0 (0.00)	0 (0.00)	1.083	0.606
Natural breeding	72 (100)	34 (100)	32 (100)	138 (100)		
Total	72 (100)	34 (100)	32 (100)	138 (100)		

Ram selection for breeding						
Select best ram for mating	72 (100)	34 (100)	27 (84.38)	133 (96.38)	11.82	0.001
No ram selection practice	0 (0.00)	0 (0.00)	5 (15.62)	5 (3.62)		
Total	72 (100)	34 (100)	32 (100)	138 (100)		
Source of breeding rams					Chi‐square	Asymptotic *p* value
Born in the flock	17 (23.61)	7 (20.59)	3 (9.38)	27 (19.56)	10.47	0.106
Neighbors	12 (16.67)	8 (23.53)	5 (15.63)	25 (18.12)		
Purchased from market	6 (8.33)	3 (8.82)	9 (28.13)	18 (13.04)		
Both neighbor and born in the flock	37 (51.39)	16 (47.06)	15 (46.88)	68 (49.28)		
Total	72 (100)	34 (100)	32 (100)	138 (100)		

Selecting criteria for breeding rams						
Body conformation	24 (33.33)	11 (32.35)	12 (37.50)	47 (34.06)	3.348	0.911
Age	21 (29.17)	9 (26.47)	7 (21.88)	37 (26.81)		
Meat production potential	15 (20.83)	6 (17.65)	5 (15.63)	26 (18.84)		
Pedigree	7 (9.72)	5 (14.71)	3 (9.38)	15 (10.87)		
Coat color	5 (6.94)	3 (8.82)	5 (15.63)	13 (9.42)		
Total	72 (100)	34 (100)	32 (100)	138 (100)		

The present findings were consistent with the observations of Debir and Amelmal [9], who reported that farmers in the Gedeb District of the Gedeo Zone, Southern Ethiopia, rarely used a breeding ram purchased from the market. On the other hand, standing against the remarks of the present study, the same authors highlighted that rams from the neighbors were the main breeding source for their flock. The preference of farmers toward ram selection criteria exhibited a consistent pattern across agroecology in that priority was given to body conformation of the ram (32.4%–37.5%), followed by age (21.9%–29.2%) and meat production potential (15.6%–20.8%). Pedigree and coat color were rarely used as parameters for ram selection. In agreement with the present study, farmers in Tahtayadyabo and Kafta Humera districts of Tigray Region, Northwestern Ethiopia [27], used pedigree as the least important ram selection criteria, but coat color was considered among the priority criteria of selection, as opposed to the present study. According to Abate and Masho [5], traditional, experience‐based selection systems, while lacking formal performance records, effectively allow farmers to maintain and slowly improve flocks that are resilient, productive, and aligned with their production objectives of generating immediate income and sustaining the genetic qualities of their herds.

### 3.7. Reproductive Performances of Sheep

The reproductive performance of sheep in the study area is presented in Table 9.

**Table 9:** Productive and reproductive performance (months, mean ± SD) of sheep in the Loma District in Dawro Zone.

Variables	Agroecology			Overall	SEM	*p* value
	Highland	Midland	Lowland			
Rams′ AFS	6.81 ± 1.07^a^	6.32 ± 0.68^b^	6.22 ± 0.49^b^	6.55 ± 0.91	0.078	0.002
Ewes′ AFS	7.44 ± 1.24^a^	7.12 ± 1.18^a^	7.06 ± 1.16^a^	7.28 ± 1.21	0.103	0.229
AFL	12.49 ± 0.96^a^	12.15 ± 0.86^ab^	12.06 ± 0.84^b^	12.30 ± 0.93	0.079	0.049
LI	7.86 ± 1.35^a^	7.59 ± 1.23^a^	7.31 ± 1.12^a^	7.67 ± 1.28	0.109	0.120
ALS	1.72 ± 0.74^a^	1.62 ± 0.65^a^	1.47 ± 0.57^a^	1.64 ± 0.68	0.058	0.214
SAR	8.31 ± 0.76^a^	7.97 ± 0.90^ab^	7.81 ± 0.86^b^	8.11 ± 0.84	0.072	0.011

*Note:* Means with different superscript letters (a and b) across rows varied significantly.

Abbreviations: AFL, age at first lambing; ALS, average litter size; AP, age at puberty; LI, lambing interval; SAR, slaughter age of ram; SEM, standard error of the mean.

Rams′ AFS, AFL, and SAR per household significantly varied with varying agroecology (*p* < 0.05). Midland and lowland rams had comparable AFS values (*p* > 0.05) lower than those of highland rams (*p* < 0.01). AFL and SAR showed a consistent pattern of variability with varying agroecology such that the observations of the parameters for highland flocks were higher relative to those of lowlands (*p* < 0.05) but comparable to the records of midland flocks. On the contrary, the overall mean values of ewes′ AFS, LI, and ALS were observed to be 7.28 ± 1.21 months, 7.67 ± 1.28 months, and 1.64 lambs per parturition, respectively, with the values showing no significant variability among households irrespective of agroecological differences (*p* > 0.05). In three selected districts of West Welega Zone, Oromia Region, Ethiopia, Amanuel and Tekalign [10] reported rams′ AFS (7.84 months), ewes′ AFS (8.61 months), AFL (14.30 months), and LI (8.83 months) substantially higher than the mean values recorded for these parameters in the present study, but a lower ALS (1.19 lambs/parturition) compared with that of the present study (1.64 lambs/parturition). On the other hand, Kenfo et al. [24] reported rams′ AFS (7.07 months), ewes′ AFS (7.74 months), and AFL (12.84 months) nearly comparable to the present study, but an ALS (1.25 lambs/parturition) lower than that recorded in the present study. The observed disparity in the reproductive parameters of sheep studies by different authors in different parts of Ethiopia could be ascribed to genetic and environmental differences and nutritional qualities of feeds as well as disease or parasitic burdens in the respective study localities [10, 34, 35]. Supporting this scenario, Abate and Masho [5] highlighted that the sheep production sector of Ethiopia is underpinned by a rich diversity of indigenous sheep populations, which exhibit significant phenotypic variation in traits such as tail type, body size, and coat color, reflecting adaptation to diverse agroecological zones. The relatively early reported age at first service suggests that sheep in the study area may reach breeding age under the prevailing management conditions at comparatively young ages; however, reproductive efficiency should also be evaluated alongside conception rate, lamb survival, nutritional status, and lifetime reproductive performance.

### 3.8. Constraints of Sheep Production

The major constraints hindering production and productivity of sheep in the study area are presented in Table 10. Disease and parasites, inadequacy of technology, predators and thieves, feed and water shortage, drought, lack of credit services, lack of improved sheep breeds, weak extension services, and marketing problems were the major bottlenecks of sheep rearing. The present study revealed that, even though the effects of the stated factors were detected in all agroecologies of the study area, the nature and intensity of the effects confirmed by the farmers are not consistent across agroecological zones. The inconsistencies in disease and parasites, weak extension services, and feed and water shortage were the most severe factors, whereas lack of improved breeds and lack of credit services, along with drought, were the least important factors to the experience of the highland sheep herders. For midland and lowland herders, feed and water shortage and diseases and parasites were identified as the most serious problems, whereas marketing problems and lack of credit services were the least challenging ones. Generally speaking, disease and parasite effect was declared as the most critical factor among all, followed by feed and water shortage, weak extension services, and inadequacy of technological packages. On the other hand, lack of credit services was marked as the least important factor among others, preceded by drought and marketing problems.

**Table 10:** The major constraints and opportunities of sheep production.

Agroecology	Constraint	Ranked (*N*)									Total	Weight	Index	Rank
		1st	2nd	3rd	4th	5th	6th	7th	8th	9th				
Highland	Disease and parasites	34	16	14	8	0	0	0	0	0	72	580	0.1790	1st
	Weak extension	17	14	33	8	0	0	0	0	0	72	544	0.1679	2nd
	Feed and water shortage	8	35	15	14	0	0	0	0	0	72	541	0.1670	3rd
	Inadequate technology	13	7	10	42	0	0	0	0	0	72	495	0.1528	4th
	Marketing problem	0	0	0	0	32	18	13	9	0	72	289	0.0892	5th
	Predator and thief	0	0	0	0	16	33	14	9	0	72	272	0.0840	6th
	Lack of improved breed	0	0	0	0	13	14	34	8	3	72	242	0.0747	7th
	Lack of credits	0	0	0	0	11	7	5	30	19	72	177	0.0546	8th
	Drought	0	0	0	0	0	0	6	16	50	72	100	0.0309	9th
	Total	72	72	72	72	72	72	72	72	72		3240	1	

Midland	Feed and water shortage	12	18	4	0	0	0	0	0	0	34	280	0.1830	1st
	Disease and parasites	15	11	8	0	0	0	0	0	0	34	279	0.1824	2nd
	Weak extension	7	5	22	0	0	0	0	0	0	34	257	0.1680	3rd
	Inadequate technology	0	0	0	14	12	8	0	0	0	34	176	0.1150	4th
	Predator and thief	0	0	0	10	17	7	0	0	0	34	173	0.1131	5th
	Lack of improved breed	0	0	0	10	5	19	0	0	0	34	161	0.1052	6th
	Drought	0	0	0	0	0	0	16	10	8	34	76	0.0497	7th
	Marketing problem	0	0	0	0	0	0	8	17	9	34	67	0.0438	8th
	Lack of credits	0	0	0	0	0	0	10	7	17	34	61	0.0399	9th
	Total	34	34	34	34	34	34	34	34	34		1530	1	

Lowland	Feed and water shortage	16	10	6	0	0	0	0	0	0	32	266	0.1847	1st
	Disease and parasites	10	17	5	0	0	0	0	0	0	32	261	0.1813	2nd
	Drought	6	5	18	3	0	0	0	0	0	32	238	0.1653	3rd
	Weak extension	0	0	3	17	8	4	0	0	0	32	179	0.1243	4th
	Inadequate technology	0	0	0	8	16	5	3	0	0	32	157	0.1090	5th
	Predator and thief	0	0	0	4	5	19	4	0	0	32	137	0.0951	6th
	Lack of improved breed	0	0	0	0	3	4	16	6	3	32	94	0.0653	7th
	Marketing problem	0	0	0	0	0	0	5	18	9	32	60	0.0417	8th
	Lack of credits	0	0	0	0	0	0	4	8	20	32	48	0.0333	9th
	Total	32	32	32	32	32	32	32	32	32		1440	1	0

Overall	Disease and parasites	59	44	27	8	0	0	0			138	1120	0.18035	1st
	Feed and water shortage	36	63	25	14	0	0	0	0	0	138	1087	0.17504	2nd
	Weak extension	24	19	58	25	8	4	0	0	0	138	980	0.15781	3rd
	Inadequate technology	13	7	10	64	28	13	3	0	0	138	828	0.13333	4th
	Predator and thief	0	0	0	14	38	59	18	9	0	138	582	0.09372	5th
	Lack of improved breed	0	0	0	10	21	37	50	14	6	138	497	0.08003	6th
	Marketing problem	0	0	0	0	32	18	26	44	18	138	416	0.06699	7th
	Drought	6	5	18	3	0	0	22	26	58	138	414	0.06667	8th
	Lack of credits	0	0	0	0	11	7	19	45	56	138	286	0.04605	9th
	Total	138	138	138	138	138	138	138	138	138		6210	1	

Similar factors were reported affecting sheep production in different parts of Ethiopia, though the rank given by the farmers depending on the intensity of the factors showed a visible variability from region to region. In the Majang Zone of Gambella Region, Southwest Ethiopia, Assefa et al. [4] reported diseases and marketing problems as the most critical and least challenging factors of sheep production, respectively, which aligns with the present study. Contradicting the present study, weak extension service [27] and predators [24] were reported as the least critical factors in Tahtayadyabo and Kafta Humera districts of Tigray Region, Northern Ethiopia, and in Bensa District of Sidama Region, Southern Ethiopia, respectively. The discrepancy in the nature and intensity of factors on sheep production at different regions underlines that remedial actions and policy solutions being formulated should not be very general and should give due consideration to prevailing regional situations. Results of this study showed that farmers across all agroecological zones recognized the identified constraints, although their magnitude, severity, and specific impacts varied with agroecological conditions. This suggests that agroecology moderates how these factors affect farmers and livestock production. Consequently, a uniform intervention approach may be ineffective. Development programs, extension services, and policy interventions should therefore be designed and implemented according to the specific needs and conditions of each agroecological zone.

## 4. Conclusions

In the Loma District of Dawro Zone, Southwestern Ethiopia, the farmers were keeping their sheep for manure production, generating income, meat production, means of cash saving, and insurance against potential livelihood risks like crop failures or emergency financial needs. Natural pasture, crop residues, nonconventional feeds (*Atela*, brewery product of local drink), hay, river and roadside grazing, crop aftermath, and improved forages were the most important sheep feeds identified in the area. Natural mating was the principal breeding approach practiced by the farmers, with AI totally lacking in the area. Selection of breeding rams was widely practiced by farmers irrespective of agroecological differences, where the ram was sourced from neighbors and from the replacement flock as well. Farmers designed diverse preference criteria when selecting breeding stock and stock replacement, primarily focusing on traits such as body conformation and age while considering pedigree and coat color rarely. Disease and parasites, feed and water shortages, and weak extension services were the most severe constraints of sheep herding; marketing problems, drought, and lack of credit services were the least important factors. A sheep development program integrating feed improvement, veterinary services, farmer training, community‐based ram selection, and stronger extension support was highly recommended to overcome key production constraints while enhancing indigenous sheep productivity through farmers′ existing breeding knowledge. Furthermore, it should be noted that this study relied on cross‐sectional survey data and farmers′ self‐reported responses. Thus, future studies should employ longitudinal approaches to integrate biological and economic performance data to provide a more comprehensive understanding of the topic raised.

## Funding

No funding was received for this manuscript.

## Consent

Verbal informed consent was obtained from all participants prior to data collection after a comprehensive explanation of the study purpose, the voluntary nature of participation, and their right to decline participation or withdraw at any time. Participant confidentiality was ensured by using coded identifiers to protect their data.

## Conflicts of Interest

The authors declare no conflicts of interest.

## Data Availability

The data that support the findings of this study are available from the corresponding author upon reasonable request.
